# The Werner's Syndrome protein collaborates with REV1 to promote replication fork progression on damaged DNA

**DOI:** 10.1016/j.dnarep.2010.07.006

**Published:** 2010-10-05

**Authors:** Lara G. Phillips, Julian E. Sale

**Affiliations:** Medical Research Council Laboratory of Molecular Biology, Hills Road, Cambridge, CB2 0QH UK

**Keywords:** DNA damage tolerance, Translesion synthesis, Werner's Syndrome, PCNA ubiquitination, WRN, REV1

## Abstract

DNA damage tolerance pathways facilitate the bypass of DNA lesions encountered during replication. These pathways can be mechanistically divided into recombinational damage avoidance and translesion synthesis, in which the lesion is directly bypassed by specialised DNA polymerases. We have recently shown distinct genetic dependencies for lesion bypass at and behind the replication fork in the avian cell line DT40, bypass at the fork requiring REV1 and bypass at post-replicative gaps requiring PCNA ubiquitination by RAD18. The WRN helicase/exonuclease, which is mutated in the progeroid and cancer predisposition disorder Werner's Syndrome, has previously been implicated in a RAD18-dependent DNA damage tolerance pathway. However, WRN has also been shown to be required to maintain normal replication fork progression on a damaged DNA template, a defect reminiscent of REV1-deficient cells. Here we use the avian cell line DT40 to demonstrate that WRN assists REV1-dependent translesion synthesis at the replication fork and that PCNA ubiquitination-dependent post-replicative lesion bypass provides an important backup mechanism for damage tolerance in the absence of WRN protein.

## 1 Introduction

During DNA replication, attempts to excise and repair DNA damage are likely to result in replication fork collapse. Cells therefore usually opt to bypass damage encountered by replication forks, instead deferring repair until replication has been completed. DNA damage bypass can take two general forms [for recent reviews see [1,2]]. The most direct is translesion synthesis, in which the stalled replicative polymerases are replaced by specialised translesion polymerases whose active sites are able to accommodate adducted or distorted bases. Alternatively, cells can employ one of the recombinational modes of bypass that require an undamaged template, usually the newly synthesised daughter strand on the sister chromatid. In principle, lesion bypass can take place either at the replication fork or at post-replicative gaps created when replication restarts downstream of an arrested fork. We have recently demonstrated that, in DT40, these temporally distinct lesion bypass pathways are genetically defined by requirements for the C terminus of the Y-family DNA polymerase REV1 and the monoubiquitination of the DNA sliding clamp PCNA respectively [3]. Monoubiquitination of PCNA by the E3 ubiquitin ligase RAD18 [4–6] plays a central role in the control of DNA damage tolerance in all eukaryotes studied to date [4–8] both through controlling the recruitment of translesion polymerases and by promoting recombinational bypass. However, while more is now understood about the mechanisms that control lesion bypass pathways, little is known about the accessory factors that facilitate these processes. A number of recent lines of evidence, discussed below, suggest that WRN is potentially one such factor.

Werner's Syndrome (WS, OMIM 277700) is an autosomal recessive disorder caused by mutations in the WRN gene [9] and is characterised by a variety of disorders reminiscent of premature ageing; cataracts, type II diabetes mellitus, osteoporosis, atherosclerosis as well as a high incidence of unusual cancers. WRN encodes a member of the RecQ family of helicases [10] but also contains a ‘helicase, RNaseD, C-terminal conserved’ (HRDC) domain, which has DNA-binding activity [11,12], and 3′–5′ exonuclease activity due to a motif located near the N terminal of the protein [13].

The defects in WRN-deficient cells, which include slow growth, spontaneous genetic instability and sensitivity to a range of DNA damaging agents, arise from the involvement of WRN in a very wide range of DNA transactions including base excision repair, non-homologous end joining and transcription [reviewed in [14]]. Much evidence, however, points to its involvement in processes at or around the replication fork. Similarly to RecQ in *E. coli* [15] and Rqh1 in *S. pombe* [16], there is evidence that WRN helps to avoid recombination [17], possibly by promoting damage tolerance, the set of mechanisms that promote DNA damage bypass during replication. WRN has also been reported to play a role in homologous recombination itself at the level of the resolution of recombination intermediates [18–21].

Evidence implicating WRN in DNA damage tolerance pathways comes from a number of sources. WRN interacts functionally with the Y-family translesion polymerases η, κ and ι, stimulating their extension activity in the absence of PCNA [22]. WRN alleviates pausing of these polymerases at lesions by increasing the *v*_max_ of polymerization, hence it was proposed that it may promote the progression of replication forks at the expense of increased mutagenesis. This stimulatory effect of WRN on TLS polymerases was found to be present even in WRN mutants lacking either helicase or exonuclease activities. WRN also co-localises with pol η after UV-C irradiation, but formation of WRN foci did not depend on the presence of pol η [22] and a direct interaction between the proteins has not been shown.

Genetic studies in DT40 suggest that WRN functions in a RAD18-dependent DNA damage tolerance pathway [23] with a *wrn/rad18* double mutant exhibiting sensitivity to NQO or MMS equal to that of the *rad18* single mutant. From our previous work showing that RAD18 defines a post-replicative pathway of damage bypass [3], these data suggest that WRN too would act behind the replication fork. However, using DNA fibre labelling, WRN has been shown to be required for normal replication fork progression on DNA damaged with MMS [24], suggestive of a model in which WRN assists bypass of lesions at the replication fork. This phenotype is reminiscent of REV1-deficient cells in similar fibre labelling assays [3,25] and suggests that WRN is required at the replication fork.

To resolve the question of which of the temporally distinct bypass pathways, defined by RAD18-dependent PCNA ubiquitination and by REV1 [3], WRN is involved in we generated isogenic mutants in the chicken cell line DT40. Using a newly created *wrn* DT40 that lacks any detectable transcript, we show that WRN operates in a pathway dependent on REV1 to maintain replication fork progression following DNA damage and that RAD18-dependent post-replicative gap filling provides an important backup activity when WRN is lost.

## 2 Materials and methods

### 2.1 DT40 lines, culture and transfection

Culture and transfection of DT40 was carried out as described previously [26]. The *rev1* and *pcna*K164R cell lines have also been previously described [26,27]. The *rad18* mutants used were generated in our laboratory using constructs described by Takeda and co-workers [28].

### 2.2 WRN locus targeting construct assembly

The *WRN* targeting construct was assembled in pBluescript SK+ using a custom multiple cloning site (*Kpn*I*-Not*I*-Mlu*I*-Apa*I*-Eco*RI*-Bam*HI*-Sac*I) made by annealing and ligating the oligonucleotides cWRNMCSF [5′-CGCGGCCGCACGCGTGGGCCCGAATTCGGATCCGAGCT] and cWRNMCSR [5′-CGGATCCGAATTCGGGCCCACGCGTGCGGCCGCGGTAC]. The 2.89 kb 5′ arm of homology was amplified by PCR from DT40 genomic DNA using primer pair cWRKO5F2 [5′-CAGAGAGAATAACGCGTCATAAAGATTGTATCTAAATTTCTAGTCTTC] and cWRKO5R2 [5′-CTCAGGAAACAGCCACATACAAAAGGGCCCGTAATAGTTTCCAGTTCC], which introduce *Mlu*I and *Apa*I sites at the 5′ and 3′ ends respectively. The 5.54 kb 3′ arm of homology was amplified from genomic DNA using primers cWRKO3F1 [5′-GGTGTTTGTTTGCCTGTGGCCGGGCATGG] and cWRKO3R1 [5′-CCCATAAGCTCTGAGCTCTTGGGCCATGTTG] which introduce a *Bam*HI site at the 5′ end of the arm. An endogenous *Sac*I site is present at the 3′ end. The 5′ arm was inserted into the modified pBluescript first, followed by the 3′ arm and then an antibiotic resistance cassette (either neomycin, puromycin, blasticidin or histidinol) was introduced into the *Bam*HI site. The neomycin, puromycin and blasticidin resistance markers are removable using Cre recombinase as they are flanked by modified LoxP sites [29].

### 2.3 Confirmation of WRN disruption

**Fig. 1:**
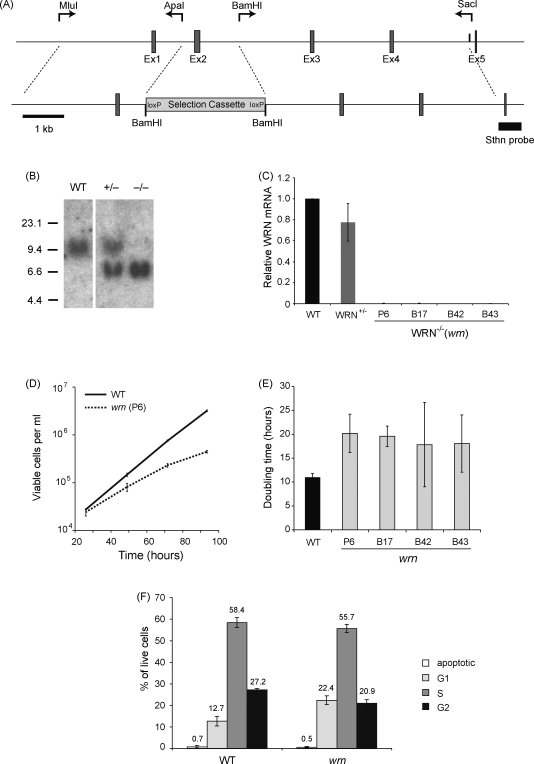
Generation of a WRN null DT40 line. (A) Schematic of the *WRN* gene targeting strategy. The binding sites of the primers used to generate the 5′ and 3′ arms of the targeting construct are shown above the locus map, along with the restriction sites used in their cloning. A selection cassette replaces exon 2 of the WRN transcript and which, following targeted integration, is predicted to lead to an out of frame splice event between exons 1 and 3 resulting in a stop codon 16 amino acids into exon 3. The Southern probe spanning exon 5 is indicated. (B) BamHI digested genomic DNA from wild type (WT), WRN^+/−^and WRN^−/−^(*wrn)* cells probed as indicated above. All lanes are from the same blot, but intervening lanes have been cut out between WT and +/−. (C) qPCR for exons 19–23 confirming loss of *WRN* transcript downstream of the disruption in four clones. (D) Growth of wild type (WT) and *wrn* clone P6. The graph represents three independent experiments with the error bars indicating the standard deviation. (E) Comparison of the doubling time in four independent clones of *wrn* DT40. (F) Cell cycle distribution of cells in asynchronous cultures of wild type and *wrn* lines. The mean percentage of 3 independent determinations is shown and the error bars represent one standard deviation.

After targeting of the first and second alleles, disruption was confirmed by Southern blot probing from the 3′ end (Fig. 1A and B). Probe DNA for Southern blotting was made by amplification of DT40 genomic DNA using the primers cWRKOPF1 [5′-GTTAATACCGTGGCTTGCTGAAGCATTTCTTGAC] and cWRKOPR2 [5′-GCTCAGCTGTAGGCTTTTGTTTTAATGAACACAAC], cloned into pCR 2.1-TOPO then excised as a *Spe*I*, Xho*I fragment. Genomic DNA from transfected clones was digested with *Bam*HI before Southern blotting.

Loss of WRN transcript was also confirmed by qPCR, carried out using a 7900HT Fast Real-Time PCR System (Applied Biosystems). Reactions were performed in MicroAmp fast optical 96-well reaction plate (Applied Biosystems) sealed with MicroAmp Optical Adhesive Film (Applied Biosystems) using SYBR GreenER qPCR SuperMix Universal (Invitrogen) and ROX reference dye. All reactions were carried out using cDNA template with primers at 400 μM. Primers to amplify WRN cDNA were designed to hybridize to the exons 19–20 and exons 22–23 junctions (Exon1920F has sequence 5′-GCGGGATGAAATCCAGTGTGTTGTGG and Exon2322R has sequence 5′-GAGGTTGCCAAACAAGAGGTTCTGCACCTG) Relative amounts of transcripts were determined using comparative quantitation relative to wild type, using β-actin as the normaliser. Primers used to amplify β-actin were BActinF1 [5′-GAGAGAAGATGACACAGATCATG] and BActinR1 [5′-GACTCCATACCCAAGAAAGATGG].

### 2.4 Measurement of growth kinetics

Cells were diluted to a density of 1 × 10^4^/ml in medium and incubated (37 °C). Viable cells were counted using a Vi-CELL cell viability analyser (Beckman Coulter) at 24 h intervals for 96 h.

### 2.5 Cell cycle analysis

Cell cycle analysis was performed as previously described [30]. Incorporated 5-bromo-2′deoxyuridine was stained using a 1:200 dilution of rat anti-BrdU antibody (BD Biosciences) followed by a 1:100 dilution of FITC-conjugated goat anti-rat antibody (BD Biosciences). FACS analysis was carried out using a FACScalibur flow cytometer (Beckman Coulter) and FlowJo 8.5.2 software (Tree Star/Stanford University).

### 2.6 Post-replication repair assay

Post-replication repair assays were carried out as previously described [3].

### 2.7 Colony survival assays

Colony survival assays were performed as previously described [31].

### 2.8 DNA fibre spreading and labelling

DNA fibre spreading and labelling was carried out as described [3]. 100 doubly labelled fibres were counted for each condition. These were derived from c. 8 slides made from two independent labelling experiments.

## 3 Results and discussion

### 3.1 Generation of a completely null wrn DT40 line

For this study we wished to produce a completely null *wrn* DT40 cell line, since an earlier *wrn* DT40 mutant retains a substantial transcript (2.5 of 4.5 kb) as assessed by Northern blot [32]. To achieve this we replaced exon 2 in both alleles of the DT40 *WRN* gene with a selectable marker by homologous recombination (Fig. 1A and B). Splicing of exon 1 to exon 3 results in an out of frame transcript and introduces a stop codon 16 amino acids into exon 3. qPCR corresponding to exons 19–23 on cDNA from the targeted clones confirmed the success of this strategy with no transcript detectable (Fig. 1C).

### 3.2 The phenotype of wrn DT40 resembles that of WS patient cells

**Fig. 2:**
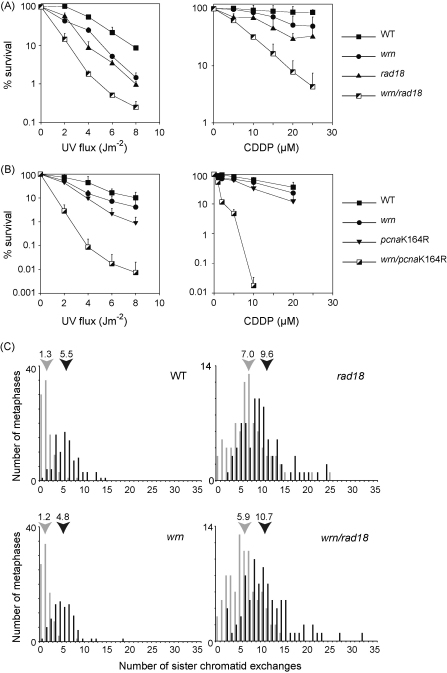
WRN and PCNA ubiquitination act in separate pathways. (A) Epistasis analysis of WRN and RAD18 for survival following treatment with 254 nm UV light (UV) and cisplatin (CDDP). (B) Epistasis analysis of WRN and cells carrying a point mutation of PCNA at K164, which prevents ubiquitination, for survival following treatment with 254 nm UV light (UV) and cisplatin (CDDP). Error bars represent the one standard deviation from the mean of three independent experiments. For clarity only the positive error bar is shown. C. Sister chromatid exchange in *wrn* and *rad18* cells. SCE with (black bars) and without (light grey bars) treatment with 0.2 ng/ml NQO. The histogram indicates the percentage of metaphases with the number of SCE indicated on the *X*-axis. The mean number of SCE in each case, derived from blind scoring of at least 100 metaphases, is indicated above the histogram. For both untreated and NQO-treated cases, the difference between wild type and *rad18* SCE is statistically significant (unpaired *t*-test, *p* < 0.0001). There is no significant difference between wild type and *wrn* or *rad18* and *wrn/rad18*.

Our *wrn* DT40 clones grew slowly, as has been reported for human WS fibroblast lines [33]. We initially characterised four independent *wrn* clones, all of which showed a comparable reduction in doubling time. For example clone P6 had doubling time at 37 °C of 20.2 ± 4.0 h compared to 11.0 ± 0.8 h for the parental wild type, measured concurrently (Fig. 1D and E). Similar to human WS fibroblasts [reviewed in [34]], this was largely explained by a prolonged cell cycle rather than an increase in spontaneous apoptosis (Fig. 1F). The increase in the proportion of G1/G0 cells in an asynchronous culture of the *wrn* mutant to 22.7%, from 12.7% in wild type, corresponds to *wrn* cells spending an average of 4.5 h in G1 as opposed to 1.4 h for wild type (Fig. 1F). Further, S-phase was also prolonged from 6.4 h in wild type to 11.3 h in *wrn* cells, again in agreement with studies of human WS cells [33,35,36]. Similar to human WS fibroblasts [37–42], *wrn* DT40 were modestly sensitive to a number of agents expected to create replication blocks including ultraviolet light [D_10_ 8.3 vs. 4.8 J/m^2^, for wild type (Fig. 2A)], the adduct/cross-linking agent cisplatin (CDDP) [D_10_ 34.1 vs. 45.7 μM (Fig. 2A)], methylmethane sulphonate (MMS) [D_10_ 99.8 vs. 142.1 parts per million (data not shown)] and nitroquinoline-1-oxide (NQO) [D_10_ 23.7 vs. 41.5 nM (data not shown)].

### 3.3 Complete WRN disruption is not epistatic with defective PCNA ubiquitination

Previous work has suggested that WRN functions in a RAD18-dependent damage avoidance pathway [23]. We re-examined this relationship by disrupting the *WRN* loci using our construct in *rad18* [28] and *pcna*K164R [27] DT40 lines. Epistasis analysis for sensitivity to 254 nm UV light and cisplatin (CDDP) showed that both *wrn/rad18* and *wrn/pcna*K164R double mutants were markedly more sensitive than either of their respective single mutants (Fig. 2A and B), showing that WRN acts largely independently of PCNA ubiquitination and, indeed, suggests that PCNA ubiquitination plays an important role in maintaining the viability of *wrn* cells following genotoxic stress.

A striking feature of *rad18* mutant lines is elevated spontaneous sister chromatid exchange [28,43]. This arises from a combination of channelling DNA lesions away from RAD18-dependent DNA damage tolerance pathways to classical homologous recombination and from a role played by RAD18 in recombination itself [44] that is independent of PCNA ubiquitination [6]. In contrast, it has been reported that inactivation of WRN in human cells does not result in elevated SCE [45]. As previously observed, *rad18* DT40 cells exhibit elevated levels of SCE in the absence of any exogenously applied DNA damage, with a mean of 7 per metaphase (Fig. 2C). The level of SCE rose further following treatment with NQO to 10 per metaphase (Fig. 2C). However, in agreement with studies in human WS cells [45], *wrn* DT40 do not exhibit elevated spontaneous SCE or an exaggerated SCE response following exposure to NQO and the *wrn/rad18* double mutant behaves the same as the *rad18* single mutant within the sensitivity limit of this assay (Fig. 2B).

### 3.4 WRN is not required for post-replicative gap filling

**Fig. 3:**
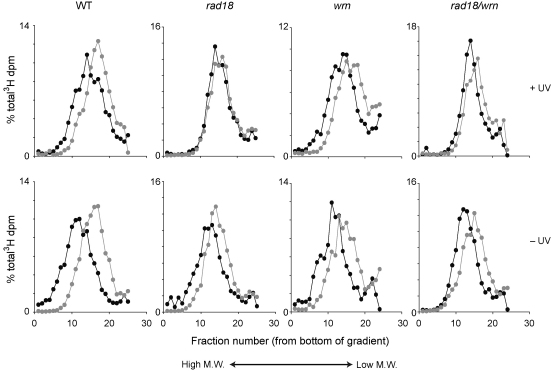
WRN is not required for post-replicative gap filling. Upper panels: Wild type, *rad18*, *wrn* and *rad18/wrn* cells were irradiated with 4 J m^−2^ 254 nm light, pulse labelled with [3H]-thymidine for 20 min and either lysed immediately (grey lines) or chased for 90’ in medium containing 10 μM thymidine before lysis (black lines). Lower panels: sham-irradiated cells.

In DT40, RAD18-mediated PCNA ubiquitination controls DNA damage bypass at post-replicative gaps that are formed in consequence of replication fork arrest with downstream resumption of replication [3]. Thus, when PCNA ubiquitination is defective the rate at which single stranded gaps are filled is reduced, i.e. PCNA ubiquitination mutants exhibit defective post-replication repair. A key prediction of the notion that WRN functions in a RAD18-dependent damage avoidance pathway [23] is that WRN should also operate behind the fork. This can be monitored by following the incorporation of a tract of tritiated label into high molecular weight single stranded DNA by velocity sedimentation on an alkaline sucrose gradient [46]. We observed, as previously reported [3,47] that *rad18* cells exhibit delayed gap filling (Fig. 3). However, such a defect is not seen in *wrn* cells and the defect in the *wrn/rad18* line is comparable to that seen in the *rad18* single mutant (Fig. 3). This suggests that WRN is not essential for timely post-replicative gap filling and together these data do not support WRN acting in the PCNA ubiquitination-dependent post-replicative DNA damage tolerance pathway.

Our conclusions are, on the face of it, in marked contrast to those reached by Dong et al. [23]. We believe that the discrepancy likely arises from the incomplete inactivation of WRN in the DT40 mutant used in the earlier study [32]. Although the targeting approached used in the work of Dong et al. will inactivate the helicase domain, most cases of WS are a result of complete functional inactivation of the gene. Loss of just the helicase activity of WRN results has been previously shown to result in a distinct and milder phenotype compared with complete inactivation of the protein [21]. Thus, while we cannot exclude that the helicase activity of WRN participates in PCNA ubiquitination-dependent damage avoidance, our results clearly demonstrate that the complete WRN protein fulfils an important function in the absence of PCNA ubiquitination. To further address this issue, we have tried extensively to perform complementation of our *wrn* mutant. However, we have been unable to obtain clones ectopically expressing the WRN protein despite using a number of different promoter systems. It will therefore be necessary to further these studies by creating endogenous mutations in the *WRN* locus.

### 3.5 WRN is required to maintain replication fork progression on damaged DNA

**Fig. 4:**
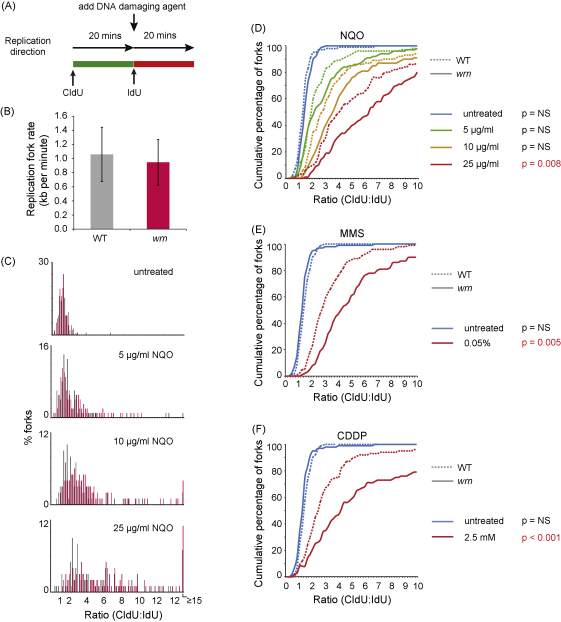
WRN is required to maintain replication fork progression on a damaged DNA template. (A) Schematic of DNA labelling experiments. Cells are pulse labelled first with chlorodeoxyuridine (CldU) for 20 min and then with iododeoxyuridine (IdU) for 20 min. DNA damage can be added along with the IdU to assess the effect on replication progression compared with the first labelling period without damage. Each period of labelling corresponds to synthesis of about 20 kb of DNA. Note the concentrations of drug used are supralethal in order to achieve a high probability of damage forming within c. 20 kb ahead of the fork [3]. (B) Fork progression on undamaged DNA in *wrn* cells is comparable to wild type (WT). Average fork rate during the first 20 min calculated using a previous calibration for this method [51] from 100 doubly labelled fibres for each case. The error bars indicate one standard deviation. There is no significant difference between WT and *wrn* (unpaired *t*-test). (C) Histograms of DNA fibre CldU:IdU ratios showing replication stalling in response to increasing doses of 4-nitroquinoline-1-oxide (NQO). Wild type: grey bars; *wrn*: red bars. (D–F) Cumulative percentage plots of DNA fibre CldU:IdU ratios. WT: dashed lines; *wrn*: solid lines. (D) 4-nitroquinoline-1-oxide (NQO). (E) Methylmethane sulphonate (MMS). (F) Cisplatin (CDDP). Statistical significance was assessed using the two-sample Kolmogorov–Smirnov (K–S) test and the probability that there is no difference between WT and *wrn* at each dose is indicated.

Recent data from WRN-depleted human cells have implicated WRN in maintaining replication fork progression on a damaged DNA template [24]. Fork progression is monitored by labelling DNA *in vivo* with halogenated nucleotides and then spreading the DNA fibres on a glass slide. The tracts of labelled DNA are revealed by denaturation and staining with antibodies that recognise halogenated bases. In our version of this assay [3], we sequentially labelled cells with chlorodeoxyuridine (CldU) and iododeoxyuridine (IdU). During the second labelling period we damaged the DNA such that the advancing fork will encounter DNA damage and may stall (Fig. 4A). We can then monitor, in a large number of fibres, the length of tract replicated before and after the application of DNA damage.

In the absence of DNA damage the replication rate of *wrn* cells was comparable to wild type at around 1 kb/min (Fig. 4B). To assess the effect of disruption of WRN on fork progression following DNA damage we performed titrations in which increasing doses of DNA damaging agent were added during the second labelling period. Damage arresting replication during the second labelling period will result in shortening of the IdU labelled tract and an increase in the ratio of CldU:IdU tract lengths. As the position of the stall is random, this ratio increases stochastically resulting in both an increase in the mean CldU:IdU ratio and in the spread of values (Fig. 4C). A convenient method to represent this data is as a cumulative percentage of forks at each ratio (Fig. 4D–F). For all doses of NQO, methyl methane sulphonate and cisplatin *wrn* cells (solid lines) exhibited a more profound inability to maintain replication fork progression on a damaged template compared with wild type (dashed lines), in agreement with the recent work on human cells [24].

### 3.6 WRN functions at the replication fork to facilitate REV1-dependent translesion synthesis

**Fig. 5:**
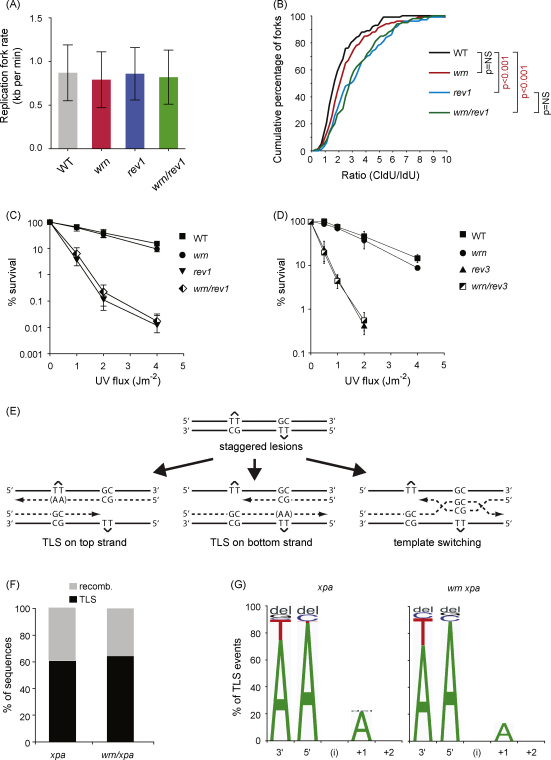
WRN acts in a REV1-dependent pathway to promote replication fork progression on damaged DNA. (A) Fork progression on undamaged DNA in wild type (WT), *wrn*, *rev1* and *wrn/rev1* cells. There is no significant difference between any of the lines (unpaired *t*-test). (B) Cumulative percentage plots of DNA fibre CldU:IdU ratios with 10 μg/ml NQO added during the second (IdU) labelling period for wild type (black line), *wrn* (red line), *rev1* (blue line) and *wrn/rev1* green line. Statistical significance assessed by the two-sample K-S test is indicated. (C and D) Epistasis analysis of WRN and REV1 (C) and REV3 (D) for survival following treatment with 254 nm UV light. E. Summary of the replicating plasmid assay for lesion bypass. For full details see Szüts et al. [50]. Briefly, the plasmid pQT_S_, which can support replication in DT40, contains staggered T–T (6-4) photoproducts placed opposite a GpC mismatch, since this is an uncommon insertion opposite this lesion. This arrangement allows determination of TLS and recombinational bypass by template switching. Since GpC opposite the lesion would also arise as a result of nucleotide excision repair, the assay is carried out in an *xpa* background. (F) The proportion of TLS vs. error-free bypass in pQTs sequences recovered from *xpa* (*n* = 130) and *xpa/wrn* cells (*n* = 70), shown as percentage of the total. (G) The pattern of nucleotide incorporation opposite the T–T (6-4) photoproduct. The percentage of each nucleotide incorporated at each position is indicated by the size of the letter of the nucleotide in the column; del: deletion.

This phenotype is strongly reminiscent of cells lacking REV1 [3,25]. REV1 plays a crucial role in coordinating translesion synthesis at the replication fork by coordinating, through its C terminus, interactions between PCNA and translesion polymerases [3,48,49]. We therefore wished to know whether this phenotypic similarity between WRN and REV1 was a reflection of their operating in common damage bypass pathway at the replication fork. To do this we created a *wrn/rev1* double mutant. In the absence of damage these cells show no defect in DNA replication rate on undamaged DNA (Fig. 5A). To examine the effect of DNA damage we chose a dose of NQO (10 μg/ml) at which we would expect to be able to readily detect an additive effect of the double disruption (see Fig. 4D). In a side-by-side comparison, *rev1* cells exhibited a more profound defect in fork progression following damage than *wrn* cells (Fig. 4B), but importantly the double *wrn/rev1* mutant was no worse than *rev1* on its own. This suggests that WRN operates at a subset of replication impediments at the fork that require REV1 for their bypass or resolution.

We next examined the genetic relationship between WRN and REV1 and REV3 for sensitivity to UV light. These experiments revealed a clear epistatic relationship between WRN and REV1/3-dependent translesion synthesis, the double mutants exhibiting no additional sensitivity to the single TLS mutants (Fig. 5C and D). This is in marked contrast to the relationship between WRN and PCNA ubiquitination (Fig. 2B). Together, these data suggest that WRN and REV1 can operate in a common pathway for the alleviation of replication arrest.

We have previously shown that REV3 is essential for translesion synthesis across (6-4) T–T photoproducts, a major UV-induced DNA lesion, *in vivo* in a replicating plasmid assay that can monitor the relative use of TLS and recombinational bypass (Fig. 5E) [50]. We also showed that loss of REV1 not only diminished the frequency of use of TLS but also resulted in reduced TLS frame fidelity. To examine the impact of the *wrn* mutation in this assay, we inactivated nucleotide excision repair, as previously described, by disrupting the *XPA* locus [50]. Since the lesion is placed opposite a G–C mismatch, this allows the distinction of recombinational bypass from excision repair. Interestingly, *wrn/xpa* cells exhibited a very similar frequency of TLS usage (Fig. 5E) and no alteration in the mutation spectrum created by the use of TLS compared with *xpa* cells (Fig. 5F). This suggests that WRN does not directly influence the TLS reaction itself or impact mutagenesis, at least in this context. Rather it appears to be required for efficient use of TLS at a subset of events at the fork. It will ultimately be interesting to determine the circumstances under which WRN is required. Nonetheless, in the absence of WRN, we suggest that bypass is more likely to take place behind the fork explaining the marked reliance of *wrn* mutant cells on PCNA ubiquitination.

## Conflicts of interest

The authors declare that there are no conflicts of interest.
